# Extensive Fracture of the Skull and Effusion of Brain, with Recovery

**Published:** 1872-02-01

**Authors:** W. W. McMann

**Affiliations:** Gardner, Ill.


### EXTENSIVE FRACTURE OF THE SKULL AND EFFUSION OF BRAIN, WITH RECOVERY.

BY W. W. MCMANN, M.D., OF GARDNER, ILL.

I was summoned in great haste, on the 10th of April, 1859, to see the son of Wm. H——, of this village, a boy some eight years of age, who unfortunately had his head crushed between the box of a wagon and a building. The injury was so severe that one ounce of brain was pressed from the wound, which was situated about half an inch above the left supraorbital ridge; this I carefully gathered up and weighed. Upon examination I discovered that it would be necessary to trephine and elevate the depressed portion of bone over the left temple. For this purpose I made a V shaped incision, raised the flaps, and sawed off an angle of bone. In the process of elevating the fractured os frontis that was driven into the brain, I scooped out one half ounce more of brain, by weight, making in all one and a half ounces. Although the fractured portion of the skull was pushed into the brain at least half to three quarters of an inch, and the boy was perfectly unconscious from the time the injury was received until the operation was performed, so much so as to be in perfect coma, with total paralysis of all the senses, still, as soon as the depression was relieved, consciousness began to return, and in two hours time he had so far recovered as to recognize his mother. From this the little patient rapidly convalesced, and in three weeks was playing about the house.

Remarks.—The above unique case was one of considerable interest to me, from the fact of the great loss of brain, and the quick recovery that followed. Also from the fact that he perfectly recovered all his mental faculties. Although nearly three years have past since the injury was received, yet we find him well and hearty, and he is considered the sharpest child in the family of six children.

---

A New Medical Baronet.—The honor of baronetcy has been conferred on the celebrated English pathologist and surgeon, Jas. Paget.

